# NanoBERTa-ASP: predicting nanobody paratope based on a pretrained RoBERTa model

**DOI:** 10.1186/s12859-024-05750-5

**Published:** 2024-03-21

**Authors:** Shangru Li, Xiangpeng Meng, Rui Li, Bingding Huang, Xin Wang

**Affiliations:** College of Big Data and Internet, Shenzhen Technology University, Shenzhen, China

**Keywords:** Nanobodies, RoBERTa, Pretrained model, prediction of binding sites, Antibody engineering, Transfer learning, Transformers

## Abstract

**Background:**

Nanobodies, also known as VHH or single-domain antibodies, are unique antibody fragments derived solely from heavy chains. They offer advantages of small molecules and conventional antibodies, making them promising therapeutics. The paratope is the specific region on an antibody that binds to an antigen. Paratope prediction involves the identification and characterization of the antigen-binding site on an antibody. This process is crucial for understanding the specificity and affinity of antibody-antigen interactions. Various computational methods and experimental approaches have been developed to predict and analyze paratopes, contributing to advancements in antibody engineering, drug development, and immunotherapy. However, existing predictive models trained on traditional antibodies may not be suitable for nanobodies. Additionally, the limited availability of nanobody datasets poses challenges in constructing accurate models.

**Methods:**

To address these challenges, we have developed a novel nanobody prediction model, named NanoBERTa-ASP (Antibody Specificity Prediction), which is specifically designed for predicting nanobody-antigen binding sites. The model adopts a training strategy more suitable for nanobodies, based on an advanced natural language processing (NLP) model called BERT (Bidirectional Encoder Representations from Transformers). To be more specific, the model utilizes a masked language modeling approach named RoBERTa (Robustly Optimized BERT Pretraining Approach) to learn the contextual information of the nanobody sequence and predict its binding site.

**Results:**

NanoBERTa-ASP achieved exceptional performance in predicting nanobody binding sites, outperforming existing methods, indicating its proficiency in capturing sequence information specific to nanobodies and accurately identifying their binding sites. Furthermore, NanoBERTa-ASP provides insights into the interaction mechanisms between nanobodies and antigens, contributing to a better understanding of nanobodies and facilitating the design and development of nanobodies with therapeutic potential.

**Conclusion:**

NanoBERTa-ASP represents a significant advancement in nanobody paratope prediction. Its superior performance highlights the potential of deep learning approaches in nanobody research. By leveraging the increasing volume of nanobody data, NanoBERTa-ASP can further refine its predictions, enhance its performance, and contribute to the development of novel nanobody-based therapeutics.

Github repository: https://github.com/WangLabforComputationalBiology/NanoBERTa-ASP

**Supplementary Information:**

The online version contains supplementary material available at 10.1186/s12859-024-05750-5.

## Introduction

Antibodies are vital components of the human immune system, characterized by their exceptional specificity and high affinity. They have extensive applications in disease diagnosis, treatment, and prevention. Nanobodies, a unique class of small antibody molecules, differ from conventional antibodies in that they naturally lack light chains [1]. This inherent feature renders nanobodies less prone to mutual adhesion and aggregation. However, their variable heavy chain (VHH) region exhibits structural stability and antigen binding activity comparable to that of full-length antibodies. Nanobodies are considered the smallest functional units known to bind target antigens (excluding just the CDR peptides). Nanobodies possess the advantages of both conventional antibodies and small molecule drugs [2]. Nanobodies are increasingly being recognized as a promising class of therapeutic biopharmaceuticals in the field of therapeutic biomedicine and clinical diagnostic reagents [3]. However, the design and development of nanobodies remain a challenging issue, requiring the resolution of numerous technical hurdles. One key challenge is accurately predicting the binding paratopes between nanobodies and antigens.

The antibody’s paratope is typically located within complementary determining regions (CDRs). The paratope of an antibody interacts with the antigen through non-covalent interactions such as hydrogen bonds, ionic bonds, van der Waals forces, and hydrophobic interactions. Predicting the complementarity-determining regions is a method to investigate the characteristics of antibodies and understand their specificity and selectivity. Predicting binding sites is crucial for comprehending the specificity of antibodies and the antigen recognition mechanism [4]. It provides guidance for vaccine design, drug development, and immunotherapy, making it of significant importance. Currently, the mainstream prediction methods include structure-based analysis and machine learning. Structure-based approaches utilize the three-dimensional structural information of antibodies and antigens, employing docking techniques to predict binding sites. On the other hand, machine learning leverages known antigen–antibody complexes and relevant features to construct models that learn patterns and rules of binding sites, enabling predictions for unknown antibodies. Studying the characteristics of nanobodies and accurately predicting their binding paratopes holds significant importance.

In recent years, with the advancements in artificial intelligence and deep learning technologies [5], training antibody models using large-scale antibody data has emerged as a novel approach for antibody design and optimization. Compared to traditional antibody research methods, deep learning techniques offer reduced time and cost requirements. With the assistance of computers, antibody researchers can handle larger datasets, predict the properties and functions of unknown antibodies, significantly improving the accuracy of antibody research. Currently, the mainstream methods in deep learning for antibodies are language models and graph neural network models. Graph neural network models can learn the relationships between antibody residues and represent the structure of paratopes, enabling tasks such as antibody docking, pairing, and paratope prediction [6]. Language models, on the other hand, can learn sequence data from a large volume of data, facilitating tasks such as antibody sequence generation, recovery, and paratope prediction.

In this work, we developed a training model called NanoBERTa-ASP, which achieved outstanding results of nanobody on smaller training datasets compared to other models. Our pre-training dataset consisted of approximately 31 million human heavy chain BCR sequences. The fine-tuning dataset comprised around 2200 annotated examples, including 1300 nanobody annotations and 900 antibody heavy chain annotations. NanoBERTa-ASP was built upon the model architecture of RoBERTa [7], a widely used generalized model. While RoBERTa was initially designed for handling textual data, antibody sequences are also composed of strings of amino acids. Therefore, it is feasible to apply the RoBERTa model to analyze and predict antibody data. In recent studies, researchers have successfully employed RoBERTa in the field of antibody research, achieving promising results. These endeavors demonstrate the potential utility of RoBERTa in antibody-related investigations, showcasing its effectiveness in tasks such as antibody sequence analysis, antigen–antibody interaction prediction, and other relevant studies.

## Experimental procedures

**Fig. 1:**
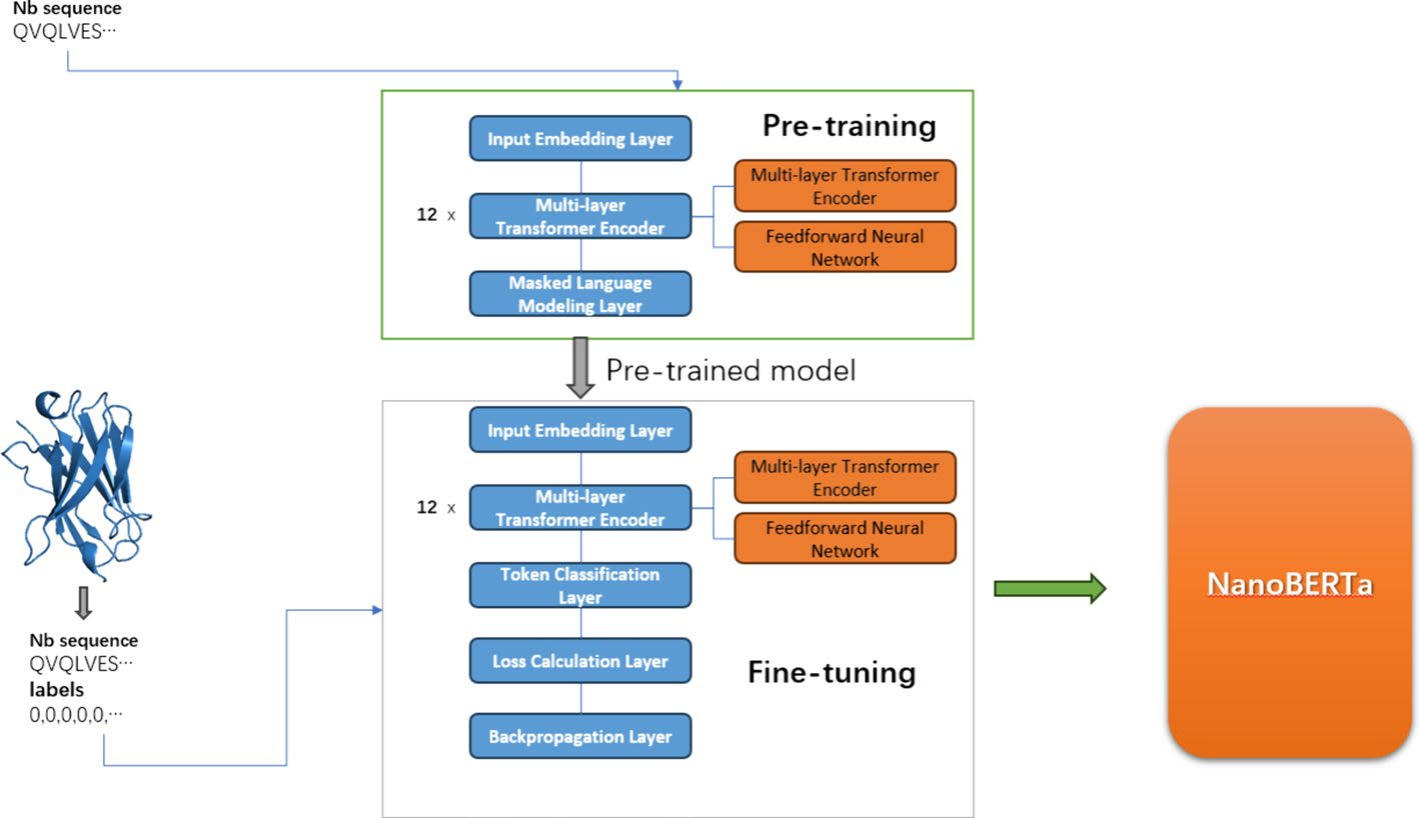
The flowchart of NanoBERTa-ASP

The flowchart of NanoBERTa-ASP was shown as Fig. 1

### Pretrain dataset

To pretrain NanoBERTa-ASP, we downloaded 70 research-based human unpaired antibody heavy chain sequences from the Observed Antibody Space (OAS) database on April 16, 2023. [8] We removed sequences containing unknown amino acids. To ensure that the model could better capture sequence features, we selected sequences with a minimum of 20 residues before the CDR1 region and a minimum of 10 residues after the CDR3 region. Subsequently, We partitioned the entire collection of 31.01 million unpaired heavy chain sequences into mutually exclusive sets for the purpose of training, testing, and validation. This was done to ensure that there was no overlap between the sequences included in each set.

The pretraining dataset consisted of approximately 24.8 million heavy chain sequences, while the pretrained validation and test sets each contained around 3.1 million heavy chain sequences.

### Finetune dataset

Due to the limited availability of nanobody data, we augmented the nanobody dataset by incorporating heavy-chain antibodies into the training set. we downloaded 7255 antibody PDB files from The Structural Antibody Database (SAbDab) on April 17th, 2023 to fine-tuning our model for binding site prediction [9]. We initially filtered 5134 crystal structures with an accuracy of 3.0 Å or higher. Structures that were defined as hapten binding in IMGT (International ImMunoGeneTics) were removed as they did not meet the binding requirements of our nanobodies. We extracted information on antibody and antigen from IMGT-numbered PDB files. Using Biopython, we constructed a NeighborSearch object based on the antigen information, which was employed to search for antibody residues adjacent to the antigen. We iterated through each nanobody residue atom's three-dimensional coordinates, using a threshold of 4.5 Å to determine whether neighboring atoms were found within the antigen.[10, 11] Residues that had antigen atoms detected within this threshold were identified as contact sites. Eventually, we selected 1070 sequences of labeled nanobodies and 4400 heavy chain sequences. To ensure that the trained model was applicable specifically to nanobodies, we only used nanobody data in both the validation and test sets. Therefore, we approximately divided the nanobody data into six equal parts (60% of all fine-tuning dataset) and selected four parts (40% of all fine-tuning dataset) for the training set. To balance the dataset, we added an equal number of heavy-chain antibody data (40% of all fine-tuning dataset) to the training set along with the nanobodies.

### NanoBERTa-ASP pre-training

NanoBETRa is a pre-trained model based on a modified version of the RoBERTa model. The vocabulary used for training consists of 24 tokens, including 20 amino acids and 4 identification tokens (<s>, </s>, <mask>, <pad>). The entire sequence is treated as a sentence, with the sequence being identified by the start token <s> and the end token </s> . The MLM(Masked Language Modeling) method was chosen for training, with 15% of the amino acids being perturbed. Similar to the RoBERTa setup, within these 15% of the amino acids, 80% of the tokens were replaced with <mask> , 10% were replaced with randomly selected amino acids, and 10% were left unchanged. During pretraining, the model predicts what kind of residue it is on the masked position. For seq in each batch, the loss is defined as:

$${\text{Loss}} = - \frac{1}{{\left| {{\text{batch}}} \right|}}\mathop \sum \limits_{{{\text{seq}}\; \in \;{\text{batch}}}} \mathop \sum \limits_{{i \in {\text{mask}}}} \log \hat{p}\left( {{\text{si}} \left| {{\text{S}}\backslash {\text{mask}}} \right.} \right)$$

${\hat{\text{p}}}\left( {{\text{si}} \left| {{\text{S}}\backslash {\text{mask}}} \right.} \right)$ represents the prediction probability of the model for sequence (s) at the i-th residual position, under the condition that the other parts of the sequence (S) masked.

### NanoBERTa-ASP fine-tuning

We consider the task of paratope prediction as a binary token classification task, where NanoBERTa-ASP predicts whether each residue in a nanobody sequence is a paratope or not. To achieve this, we add a binary classification head on top of the pre-trained model to label the sequences. During training, the model uses cross-entropy loss function to calculate the difference between the predicted probability p and the true label y, then updates the model parameters using backpropagation algorithm. The loss during fine-tuning is defined as:

$${L}_{BCE}=-\frac{1}{batch}{\sum }_{i=1}^{batch}{\sum }_{j=1}^{length}{y}_{i,j}log{p}_{i,j}+(1-{y}_{i,j})log(1-{p}_{i,j})$$

## Result

### Attention mechanism can focus on the structure of the sequence

**Fig. 2:**
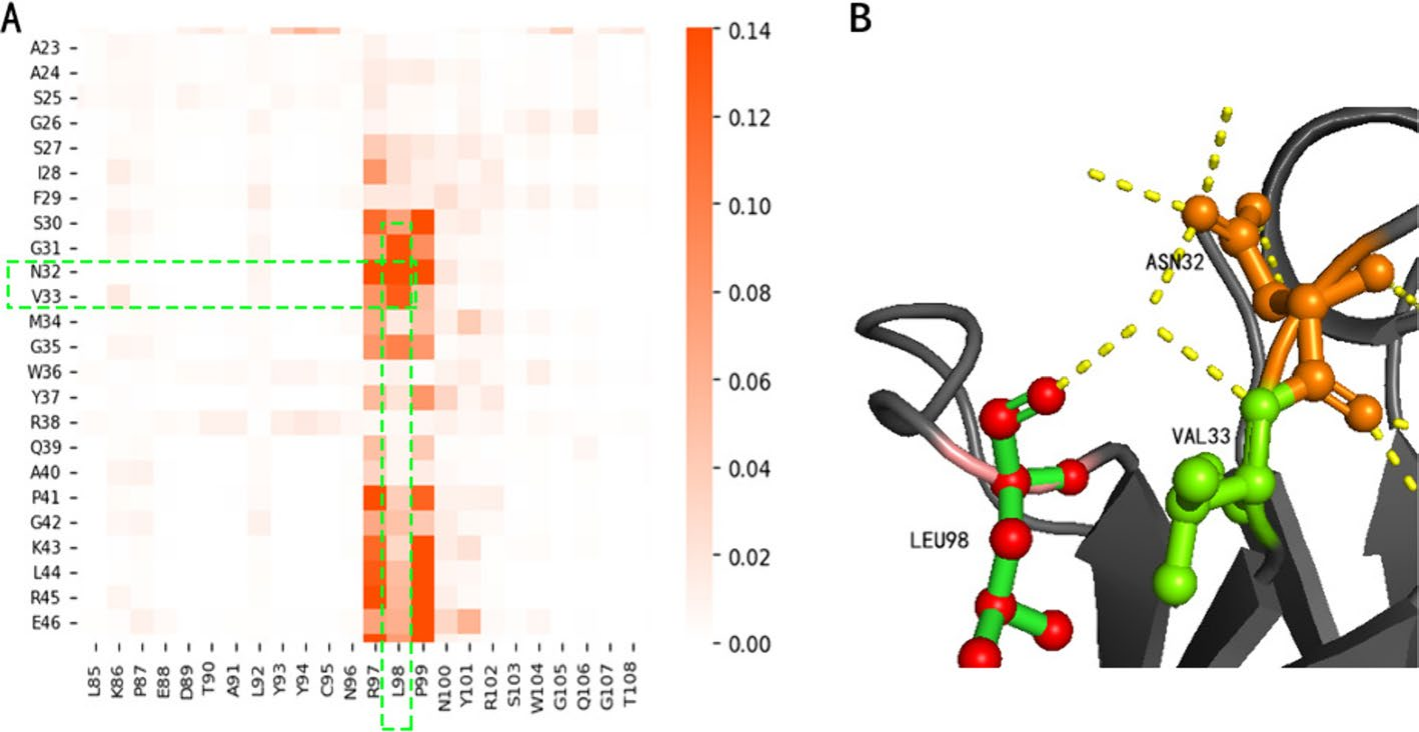
**A** Self-attention heatmap from NanoBERTa-ASP’s 12th layer, the sixth attention head for Nb-ER19 in PDB:5f7y; **B** The schematic diagram of the 3D structure of Nb-ER19 in PDB:5f7y show interaction displayed by PyMOL

As a RoBERTa-based model, NanoBERTa-ASP also habours the same multi-head attention mechanism as RoBERTa. The attention heads of NanoBERTa-ASP can focus on different parts of the sequence. NanoBERTa-ASP exhibits a higher degree of attention towards the highly variable CDR3 region. For example, when we input the nanobody Nb-ER19 into the model and output the attention layers in the form of a heatmap, we can observe that the sixth head of the twelfth layer of the model has a special attention on the positions of ASN32 and VAL33 in CDR1, LEU98 in CDR3 (PDB:5f7y [12]) (Fig. 2A). By observing in PyMOL, it was found that there was a interaction at this position (Fig. 2B), and LEU98 is also part of the paratope. This indicates that the model can learn certain structural features of antibodies through the annotated sequences.

### Performance of the NanoBERTa-ASP

**Fig. 3:**
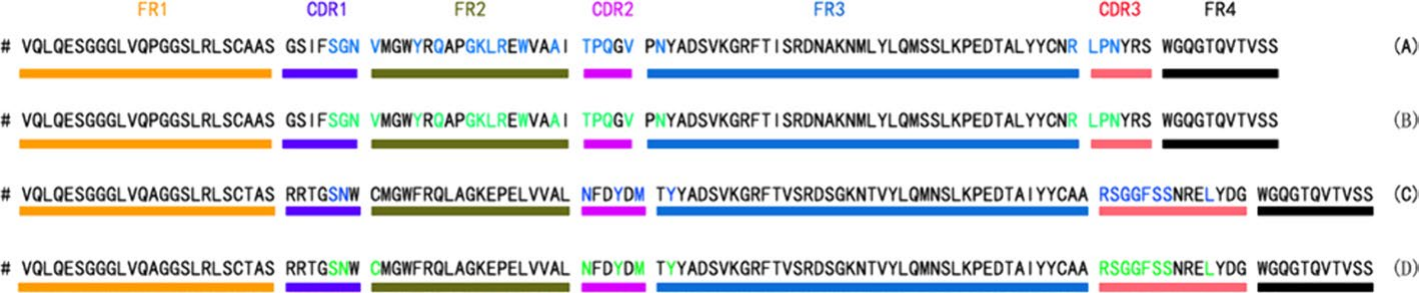
NanoBERTa-ASP accurately predicts the binding sites of nanobodies. **A** Annotated paratope positions from the crystal structures via Biopython, PDB id:5f7y; **B** Prediction of PDB id:5f7y binding sites by NanoBERTa-ASP; **C** Annotated paratope positions from the crystal structures via Biopython, PDB id:2wzp; **D** Prediction of PDB id:2wzp binding sites by NanoBERTa-ASP. Binding sites calculated by NanoBERTa-ASP are represented by green letters, and binding sites calculated by Biopython are represented by blue letters

Our model can predict binding sites in both CDR and non-CDR regions. Figure 3B and D show the predicted binding sites of NanoBERTa-ASP, compared with annotated paratope positions from the crystal structures via Biopython and PDB file shown in Fig. 3A and C. NanoBERTa-ASP can accurately identify the binding sites on the nanobody sequence, as demonstrated by comparison with the annotation from the crystal structures via Biopython and PDB file (5f7y [12] and 2wzp [13]).

To verify the generalization ability of the model, we conducted tenfold cross-validation on the model [14]. We conducted the test using two different datasets: one consisting solely of nanobody sequences for testing, and another consisting of the same number of heavy chain sequences added to the training set for training. To ensure that our evaluations were focused solely on the ability of the model to perform with respect to nanobodies, we used only nanobody sequences in our testing set. The AUC and precision obtained from the mixed dataset (AUC = 0.952, precision = 0.778) were higher than those from the pure nanobody dataset (AUC = 0.947, precision = 0.766). Through analysis of the results data, NanoBERTa-ASP shown high stability in cross-validation (Additional file 1: Fig. 2).

**Fig. 4:**
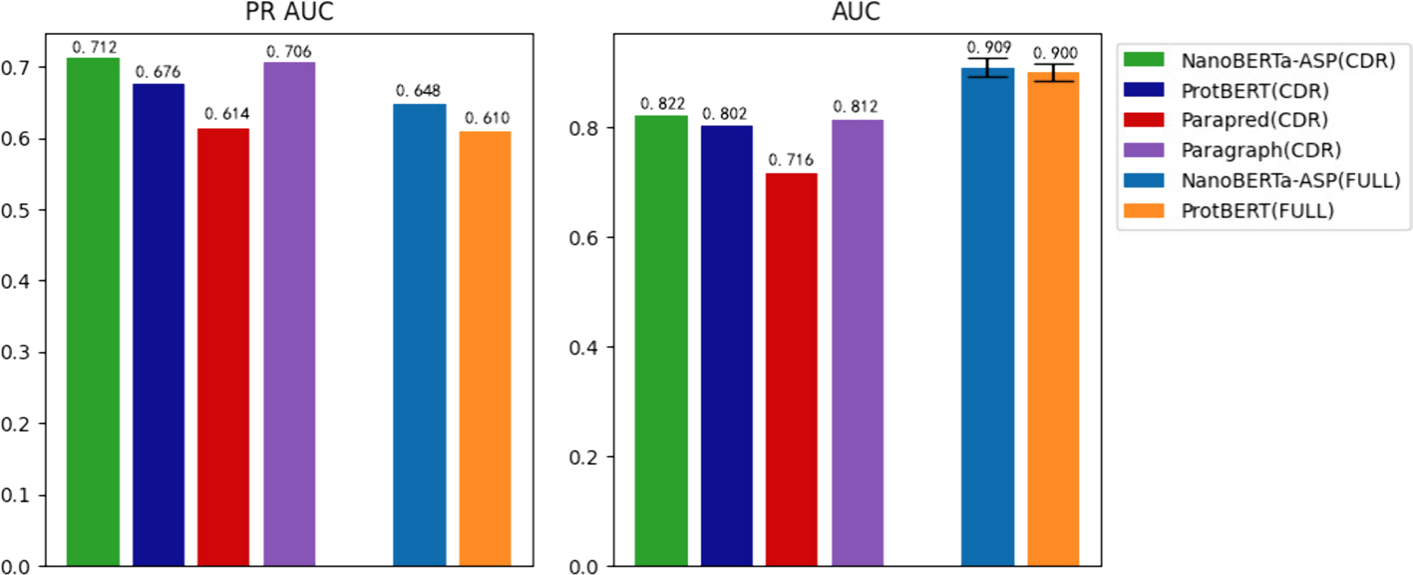
Comparison of NanoBERTa-ASP with Other Models Based on PR AUC (left) and AUC (right)

**Fig. 5:**
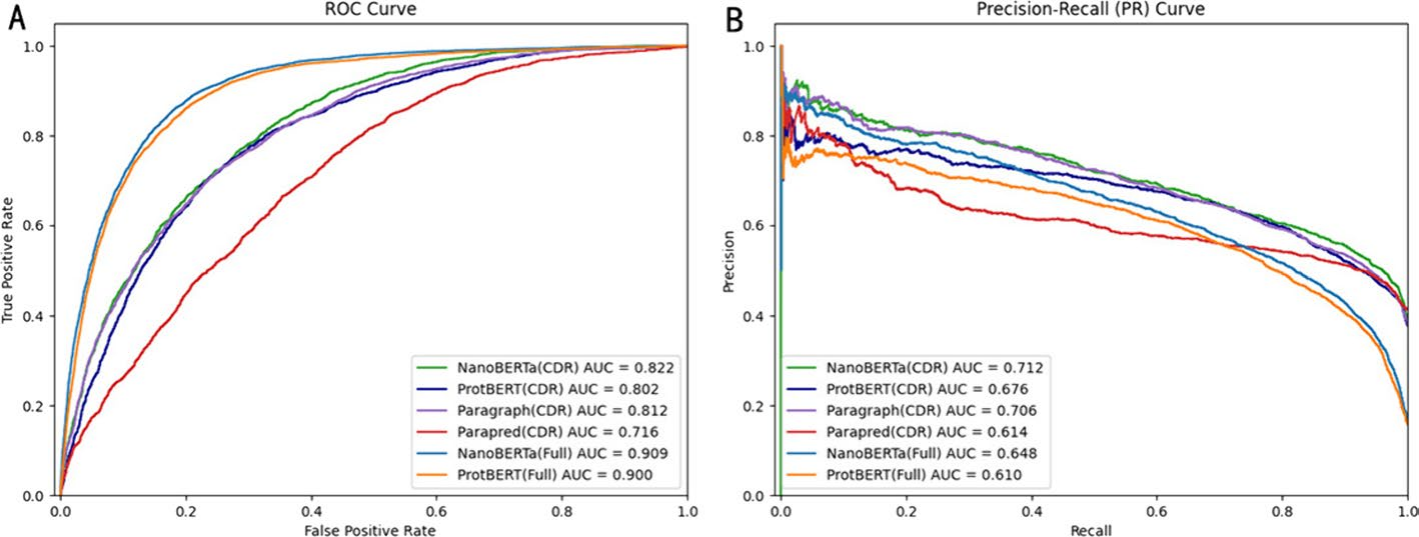
NanoBERTa-ASP outperformed other models in predicting binding sites Based on **A** Precision-Recall Curve and **B** ROC Curve

We also compared our model with currently available models for predicting binding sites, including ProtBERT, Paraperd, and Paragraph (Figs. 4 and 5). As Paraperd and Paragraph only predict binding sites in the CDR region, we extracted the CDR part of the predicted results from the complete sequence predictions of ProtBERT and NanoBERTa-ASP for comparison [15–17].

NanoBERTa-ASP exhibits superior performance compared to publicly available tools in terms of the complete sequence of nanobodies and the CDR region. As the CDR region is a highly variable region that is harder to predict, and also the main region where binding sites exist, the model is more focused on predicting positive samples, which may result in a lower AUC score for the CDR region than for the complete sequence, but a higher precision score in the CDR region (Figs. 4 and 5). Our results demonstrated that NanoBERTa-ASP exhibits exceptional performance even with limited data, highlighting its significant potential for accurately predicting binding sites of nanobodies.

NanoBERTa-ASP, trained on a dataset of 31 million heavy chains, achieved comparable performance to ProtBERT, which was trained on a much larger dataset of 217 million proteins. This suggests that NanoBERTa-ASP is an effective model for nanobody sequence analysis, even with a smaller dataset (see Figs. 4, 5 and Additional file 1: Table 1).

## Conclusion and discussion

We evaluated NanoBERTa-ASP's performance on nanobody binding site prediction and compared it to existing methods. When benchmarking, the test dataset containing only nanobody sequences was input into each model to obtain predicted binding results. PRAUC and ROC values were then calculated from these predictions and the annotated binding sites for quantitative assessment.

To enable comparison between full-sequence and CDR-only predicting models, we also extracted the CDR region predictions. As expected, ROC values were relatively higher and PRAUC values lower when evaluating full sequences compared to isolated CDRs. This is because non-CDR regions typically contain a larger proportion of negative samples (non-binding sites), introducing imbalance that impacts how ROC and PRAUC measure performance [16].

Traditionally, Area Under the Receiver Operating Characteristic Curve (AUROC/AUC) is used to evaluate prediction quality. However, in imbalanced datasets like binding site prediction where positive sites (binding sites) are the minority, Precision-Recall AUC (PRAUC) provides a more sensitive measure of how well a classifier identifies these rare cases [16]. PRAUC incorporates precision at different recall levels, emphasizing prediction of the minority class which in our case are binding sites.

Moving forward, several approaches may help further improve NanoBERTa-ASP. During pre-training, heavy chain clustering and upweighting CDR regions could enable the model to better capture nanobody characteristics. Larger datasets and batch sizes from emerging cryo-EM data may also enhance performance when training with more abundant information. Moreover, using techniques like surface plasmon resonance for precise binding site mapping could provide higher-quality annotations to train on.

In the last few years, algorithms based on BERT [17–19], RoBERTa [7, 11, 20] or graph networks [15, 16] have achieved state-of-the-art performance for various protein prediction tasks [15, 16]. While each tool has its niche, NanoBERTa-ASP excels specifically for nanobody analysis thanks to our self-supervised pre-training approach strategically developed for the nanobody domain. As nanobody datasets grow exponentially, we expect NanoBERTa-ASP's advantage over other methods will continue expanding to drive new discoveries.

In summary, NanoBERTa-ASP represents a significant advancement in nanobody binding site prediction through effective exploitation of limited data via self-supervision. Its outstanding performance demonstrates the approach's great potential for advancing computational nanobody design. NanoBERTa-ASP’s capabilities have only begun to evolve and we are confident it will continue playing an instrumental role in progressing the field.

### Supplementary Information

**Additional file 1**. Supplementary information.

## Data Availability

All scripts and datasets used can be found at https://github.com/WangLabforComputationalBiology/NanoBERTa-ASP
