# Correction: NanoBERTa-ASP: predicting nanobody paratope based on a pretrained RoBERTa model

**DOI:** 10.1186/s12859-024-05790-x

**Published:** 2024-05-14

**Authors:** Shangru Li, Xiangpeng Meng, Rui Li, Bingding Huang, Xin Wang

**Affiliations:** College of Big Data and Internet, Shenzhen Technology University, Shenzhen, China

**Correction: BMC Bioinformatics (2024) 25:122** 10.1186/s12859-024-05750-5

Following the publication of the original article [1], the authors identified that the foundation and grant number were missing from the funding statement. The correct funding is given below.

The incorrect funding is: This study was supported by the Project of the Educational Commission of Guangdong Province of China (No. 2022ZDJS113).

The correct funding is: This study was supported by the Project of the Educational Commission of Guangdong Province of China (No. 2022ZDJS113) and Natural Science Foundation of Top Talent of SZTU (Grant No. GDRC202213).

The original article [1] has been corrected.
