# Supplementary material for: NanoBERTa-ASP: predicting nanobody paratope based on a pretrained RoBERTa model
Source: BMC Bioinformatics. 2024 Mar 21;25:122. doi: 10.1186/s12859-024-05750-5 (PMC10956323; doi:10.1186/s12859-024-05750-5)
Supplement: Supplementary file 1 — Additional file 1. Supplementary information. [file 12859_2024_5750_MOESM1_ESM.docx]

Supplementary information:

1. Details of the benchmark process

To separate CDR regions, we used AbNumber’s (https://github.com/prihoda/AbNumber) IMGT scheme to separate CDR1, CDR2, CDR3 from frame FR1, FR2, FR3 and FR4.

Paragraph

The Paragraph model is based on equivariant graph neural networks. Initially, the model was trained using 205 complexes from PECAN, and later trained on a larger dataset from SAbDab. The Paragraph model was trained with 16 different random seeds, with a maximum of 300 epochs per seed. To make predictions, Paragraph requires input in the form of a PDB file and focuses on predicting the binding sites within the CDR regions.

Paragraph model was downloaded from <https://github.com/oxpig/Paragraph.> As Paragraph need the input with the coordinate information as PDB files, after this criterion, only 248 PDB files were found with sequence information. (https://github.com/WangLabforComputationalBiology/NanoBERTa-ASP/blob/main/assets/nanotest.csv)

ProtBERT

ProtBERT is a BERT based antibody language model that uses masked language modeling (MLM) for pretraining. The dataset of ProtBERT is Uniref100, consistent of 217 million protein sequences. In pretraining, the proportion of ProtBERT masks used is consistent with BERT, both being 15%.

We obtained the ProtBERT model from Hugging Face (https://huggingface.co/Rostlab/prot_bert) and fine-tuned it using the same dataset (https://github.com/WangLabforComputationalBiology/NanoBERTa-ASP/blob/main/assets/nanotest.csv). The only fine-tuning difference is that we used “RobertaTokenizer” and “RobertaForTokenClassification” while they used “BertTokenizer” and “BertForTokenClassification” besides the format difference.

Paraperd

Paraperd is a model based on CNN(convolutional neural networks) and RNN(recurrent neural networks) It used a subset of the Structural Antibody Database (SAbDab) to train and test. Paraperd trained with 277 complexes.

Paraperd provided a web service (https://www-cohsoftware.ch.cam.ac.uk/, registered and then select Paraperd) to output the binding probabilities as annotations from the protein sequence. We used the same testing dataset as above and wrote a Python script to automate Paraperd online process.

AntiBERTa

AntiBERTa is an antibody language model based on RoBERTa. Its dataset includes 42.3M heavy chain and 15.3M light chain sequences.

Because of the complete dataset and model of AntiBERTa were not provided, we did not compare it with our model in the benchmark.

AntiBERTy

AntiBERTY is a BERT based model that is pretrained on 558M natural antibody sequences. It can be used to predict the type of chain, the identity of masked residues, and calculate the pseudo log-likelihood of a sequence.

After fine-tuning our dataset, AntiBERTy's ability is similar to ours, and t-tests have shown that there is no significant difference in predicting the paratope of nanobodies. And the dataset used in NanoBERTa in the pretraining is much smaller than AntiBERTa.

we also added a benchmark ReadME file at <https://github.com/WangLabforComputationalBiology/NanoBERTa-ASP/blob/main/Benchmark/ReadME.md> with the code of benchmarking.


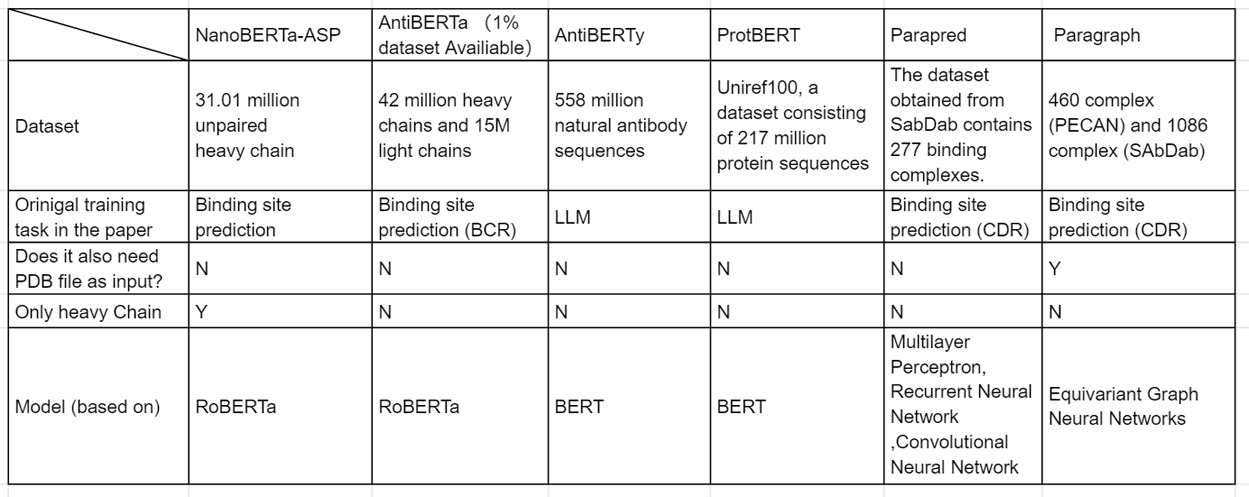


Supplementary Table 1. Comparison among related models.

1. Data preparation in benchmark

To develop a model suitable for nanobodies, we opted to utilize pure heavy chain sequences as the pre-training dataset. In order to enhance the stability of the learned sequence features, we applied specific criteria to screen the heavy chain sequences: a minimum of 20 residues before the CDR1 region and a minimum of 10 residues after the CDR3 region.

1. Validation of the differences in PR AUC and ROC among different models.


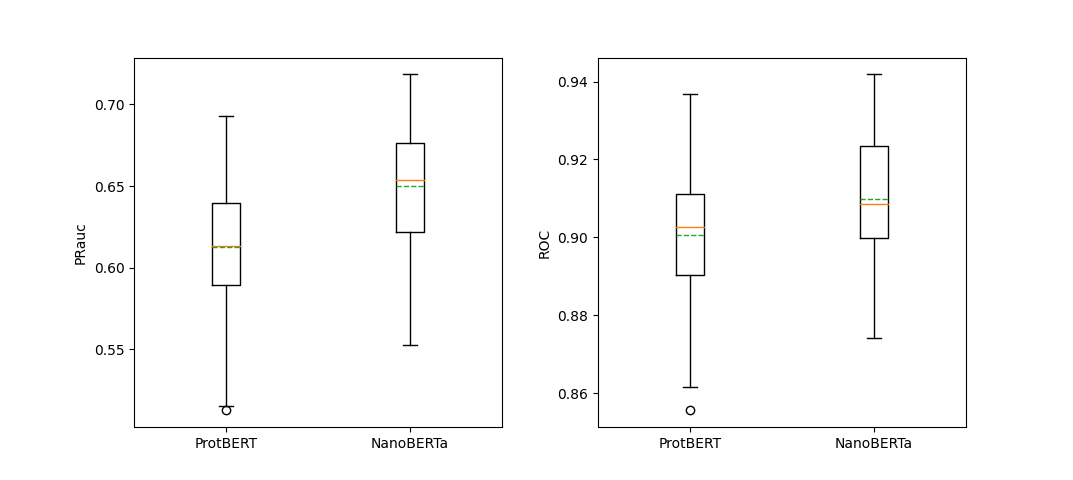


Supplementary Figure 1. T-test Result for ProtBERT and NanoBERTa (The green dashed line represents the mean, the yellow solid line represents the median).

To demonstrate the significant difference between ProtBERT model and ours, we conducted 5 rounds of 10-fold cross-validation and utilized a t-test for validation. The T-test results are as follows: P = 0.0078< 0.05, t = -2.717, indicating a significant difference.

1. 10 fold cross-validation results


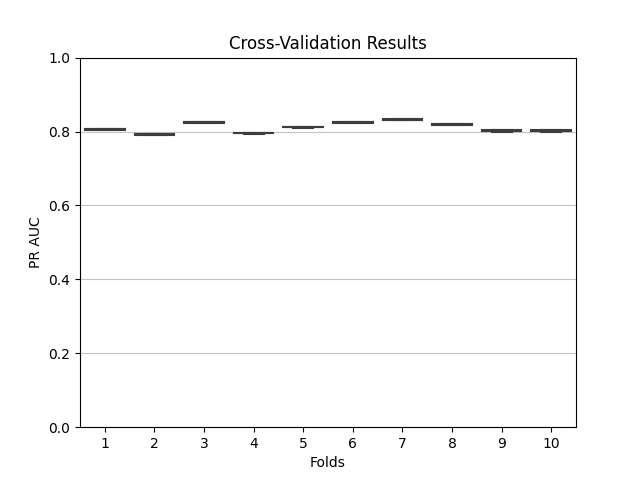


Supplementary Figure 2. 10 fold cross-validation results (The green dashed line represents the mean, the yellow solid line represents the median).

We performed 10-fold cross validation of the model. In order to verify the ability of the model to the nanobodies, we used the same way as fine-tuning to retain only VHH for the validation set. The results of the 10-fold cross validation are shown in the figure above.The datasets used for cross validation were uploaded to GitHub: <https://github.com/WangLabforComputationalBiology/NanoBERTa-ASP/blob/main/assets/10-Fold.zip> .
